# Supplementary material for: Are metabolic abnormalities the missing link between complete blood count-derived inflammatory markers and diabetic foot? Evidence from a large population study
Source: PLoS One. 2025 Jun 12;20(6):e0326082. doi: 10.1371/journal.pone.0326082 (PMC12161541; doi:10.1371/journal.pone.0326082)

A

Subgroup	Event	Subgroup1	Levels	OR.(95%CI)	P.value	P.for.interaction
**Overall**	**1246(100%)**					
**Gender**						**0.875**
Male	635(51%)		MLR_Group=Q1			
			MLR_Group=Q2	0.95(0.38−2.4)	0.915	
			MLR_Group=Q3	0.94(0.39−2.26)	0.891	
			MLR_Group=Q4	1.48(0.66−3.34)	0.344	
Female	611(49%)		MLR_Group=Q1			
			MLR_Group=Q2	0.8(0.35−1.85)	0.605	
			MLR_Group=Q3	1.27(0.58−2.74)	0.549	
			MLR_Group=Q4	1.3(0.58−2.94)	0.528	
**Race**						**0.063**
Mexican American	371(29.8%)		MLR_Group=Q1			
			MLR_Group=Q2	0.76(0.26−2.22)	0.622	
			MLR_Group=Q3	1.34(0.49−3.64)	0.564	
			MLR_Group=Q4	1.22(0.42−3.57)	0.713	
Other Hispanic	55(4.4%)		MLR_Group=Q1			
			MLR_Group=Q2	0.35(0.03−4.01)	0.401	
			MLR_Group=Q3	0(0−Inf)	0.996	
			MLR_Group=Q4	0.24(0.01−3.95)	0.315	
non-Hispanic White	488(39.2%)		MLR_Group=Q1			
			MLR_Group=Q2	2.09(0.41−10.76)	0.379	
			MLR_Group=Q3	4.23(0.94−19.12)	0.061	
			MLR_Group=Q4	4.39(0.99−19.44)	0.051	
non-Hispanic Black	286(23%)		MLR_Group=Q1			
			MLR_Group=Q2	1.39(0.47−4.07)	0.548	
			MLR_Group=Q3	0.16(0.02−1.3)	0.085	
			MLR_Group=Q4	1.06(0.32−3.55)	0.921	
Others	46(3.7%)		MLR_Group=Q1			
			MLR_Group=Q2	0(0−Inf)	0.998	
			MLR_Group=Q3	0(0−Inf)	0.998	
			MLR_Group=Q4	1.47(0.06−38.79)	0.817	
**Marry**						**0.978**
yes	735(59%)		MLR_Group=Q1			
			MLR_Group=Q2	0.83(0.37−1.88)	0.662	
			MLR_Group=Q3	0.99(0.45−2.16)	0.981	
			MLR_Group=Q4	1.29(0.62−2.71)	0.494	
no	511(41%)		MLR_Group=Q1			
			MLR_Group=Q2	0.96(0.38−2.43)	0.932	
			MLR_Group=Q3	1.21(0.52−2.84)	0.655	
			MLR_Group=Q4	1.73(0.77−3.89)	0.185	
**Hypertension**						**0.431**
Hypertension	828(66.5%)		MLR_Group=Q1			
			MLR_Group=Q2	1.19(0.57−2.5)	0.644	
			MLR_Group=Q3	1.09(0.52−2.28)	0.822	
			MLR_Group=Q4	1.73(0.87−3.44)	0.118	
Health	418(33.5%)		MLR_Group=Q1			
			MLR_Group=Q2	0.46(0.14−1.52)	0.201	
			MLR_Group=Q3	1.2(0.48−2.99)	0.7	
			MLR_Group=Q4	1.32(0.52−3.36)	0.555	
**Coronary heart disease(CHD)**						**0.025**
CHD	148(11.9%)		MLR_Group=Q1			
			MLR_Group=Q2	39328982.77(0−Inf)	0.99	
			MLR_Group=Q3	19072945.69(0−Inf)	0.99	
			MLR_Group=Q4	22956973.23(0−Inf)	0.99	
Health	1098(88.1%)		MLR_Group=Q1			
			MLR_Group=Q2	0.65(0.33−1.28)	0.213	
			MLR_Group=Q3	0.94(0.52−1.73)	0.851	
			MLR_Group=Q4	1.32(0.74−2.34)	0.344	
**Heart failure(HF)**						**0.691**
Heart failure	158(12.7%)		MLR_Group=Q1			
			MLR_Group=Q2	2.39(0.39−14.67)	0.347	
			MLR_Group=Q3	1.91(0.35−10.41)	0.455	
			MLR_Group=Q4	2.81(0.55−14.28)	0.214	
Health	1088(87.3%)		MLR_Group=Q1			
			MLR_Group=Q2	0.77(0.4−1.48)	0.43	
			MLR_Group=Q3	1(0.54−1.86)	0.995	
			MLR_Group=Q4	1.3(0.72−2.36)	0.378	
**Age**						**0.873**
Old	617(49.5%)		MLR_Group=Q1			
			MLR_Group=Q2	1.13(0.39−3.29)	0.819	
			MLR_Group=Q3	1.46(0.57−3.74)	0.427	
			MLR_Group=Q4	1.89(0.78−4.59)	0.16	
Young	629(50.5%)		MLR_Group=Q1			
			MLR_Group=Q2	0.76(0.36−1.61)	0.47	
			MLR_Group=Q3	0.94(0.44−2.02)	0.878	
			MLR_Group=Q4	1.47(0.7−3.06)	0.307	

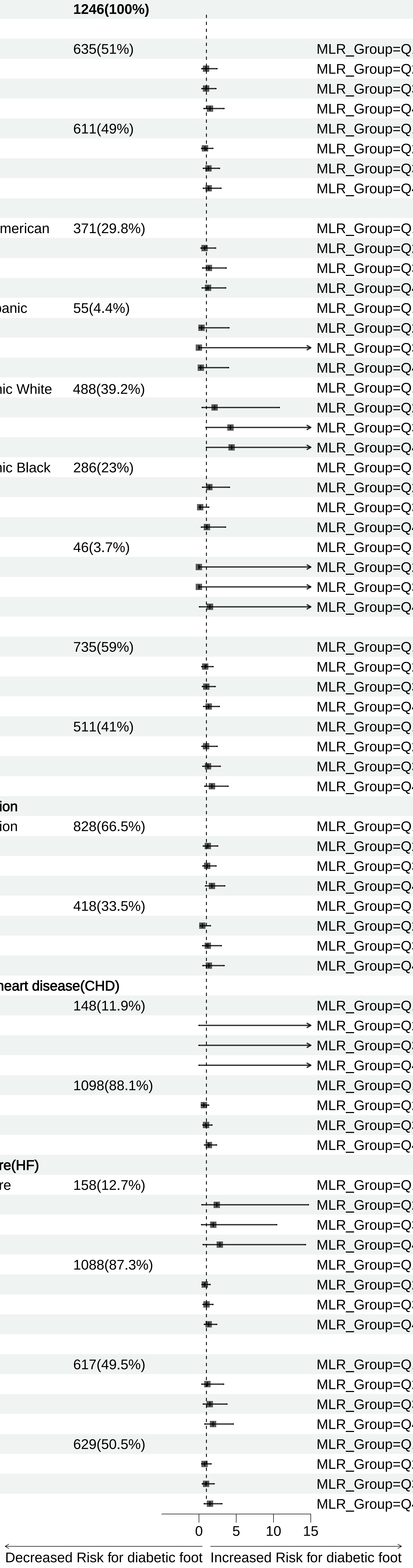

B

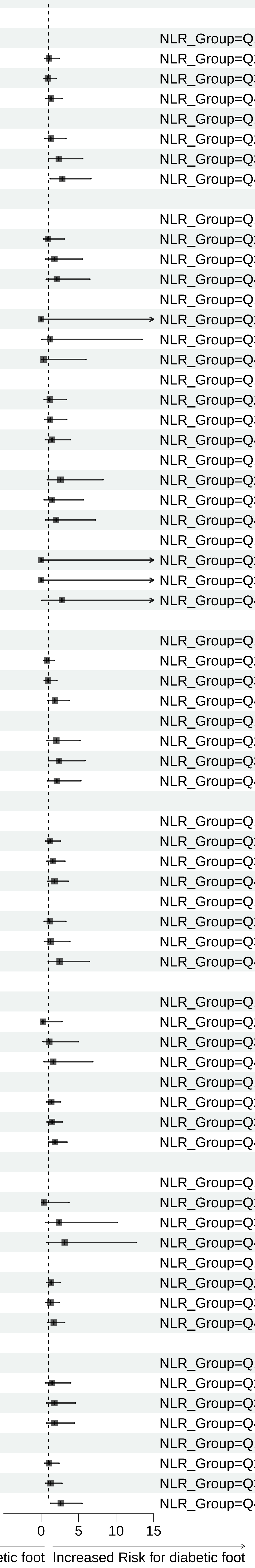

Subgroup	Event	Levels	OR.(95%CI)	P.value	P.for.interaction
**Overall**	**1246(100%)**				
**Gender**					**0.295**
Male	635(51%)	NLR_Group=Q1			
		NLR_Group=Q2	1.06(0.47−2.42)	0.889	
		NLR_Group=Q3	0.88(0.39−2.01)	0.765	
		NLR_Group=Q4	1.32(0.63−2.79)	0.465	
Female	611(49%)	NLR_Group=Q1			
		NLR_Group=Q2	1.3(0.51−3.3)	0.586	
		NLR_Group=Q3	2.35(1−5.53)	0.049	
		NLR_Group=Q4	2.82(1.2−6.63)	0.018	
**Race**					**0.544**
Mexican American	371(29.8%)	NLR_Group=Q1			
		NLR_Group=Q2	0.91(0.27−3.08)	0.877	
		NLR_Group=Q3	1.78(0.58−5.51)	0.316	
		NLR_Group=Q4	2.09(0.67−6.49)	0.201	
Other Hispanic	55(4.4%)	NLR_Group=Q1			
		NLR_Group=Q2	0(0−Inf)	0.996	
		NLR_Group=Q3	1.22(0.11−13.44)	0.873	
		NLR_Group=Q4	0.33(0.02−5.96)	0.449	
non-Hispanic White	488(39.2%)	NLR_Group=Q1			
		NLR_Group=Q2	1.16(0.4−3.36)	0.79	
		NLR_Group=Q3	1.21(0.43−3.4)	0.72	
		NLR_Group=Q4	1.46(0.55−3.91)	0.452	
non-Hispanic Black	286(23%)	NLR_Group=Q1			
		NLR_Group=Q2	2.58(0.81−8.24)	0.109	
		NLR_Group=Q3	1.48(0.39−5.62)	0.561	
		NLR_Group=Q4	2.01(0.56−7.26)	0.286	
Others	46(3.7%)	NLR_Group=Q1			
		NLR_Group=Q2	0(0−Inf)	0.999	
		NLR_Group=Q3	0(0−Inf)	0.998	
		NLR_Group=Q4	2.77(0.08−98.93)	0.577	
**Marry**					**0.247**
yes	735(59%)	NLR_Group=Q1			
		NLR_Group=Q2	0.76(0.33−1.75)	0.517	
		NLR_Group=Q3	0.93(0.41−2.09)	0.853	
		NLR_Group=Q4	1.83(0.9−3.74)	0.097	
no	511(41%)	NLR_Group=Q1			
		NLR_Group=Q2	2.03(0.79−5.17)	0.139	
		NLR_Group=Q3	2.39(0.97−5.86)	0.057	
		NLR_Group=Q4	2.1(0.83−5.3)	0.117	
**Hypertension**					**0.903**
Hypertension	828(66.5%)	NLR_Group=Q1			
		NLR_Group=Q2	1.21(0.56−2.59)	0.627	
		NLR_Group=Q3	1.56(0.77−3.17)	0.218	
		NLR_Group=Q4	1.8(0.9−3.6)	0.099	
Health	418(33.5%)	NLR_Group=Q1			
		NLR_Group=Q2	1.15(0.4−3.29)	0.8	
		NLR_Group=Q3	1.28(0.43−3.82)	0.659	
		NLR_Group=Q4	2.47(0.95−6.43)	0.064	
**Coronary heart disease(CHD)**					**0.413**
CHD	148(11.9%)	NLR_Group=Q1			
		NLR_Group=Q2	0.25(0.02−2.78)	0.258	
		NLR_Group=Q3	1.08(0.24−4.96)	0.916	
		NLR_Group=Q4	1.62(0.38−6.88)	0.515	
Health	1098(88.1%)	NLR_Group=Q1			
		NLR_Group=Q2	1.36(0.71−2.6)	0.35	
		NLR_Group=Q3	1.47(0.77−2.81)	0.238	
		NLR_Group=Q4	1.86(1.01−3.45)	0.047	
**Heartfailure(HF)**					**0.125**
Heart failure	158(12.7%)	NLR_Group=Q1			
		NLR_Group=Q2	0.36(0.03−3.7)	0.387	
		NLR_Group=Q3	2.4(0.57−10.17)	0.235	
		NLR_Group=Q4	3.13(0.77−12.71)	0.111	
Health	1088(87.3%)	NLR_Group=Q1			
		NLR_Group=Q2	1.33(0.7−2.54)	0.387	
		NLR_Group=Q3	1.25(0.65−2.41)	0.502	
		NLR_Group=Q4	1.69(0.91−3.12)	0.098	
**Age**					**0.45**
Old	617(49.5%)	NLR_Group=Q1			
		NLR_Group=Q2	1.47(0.55−3.94)	0.44	
		NLR_Group=Q3	1.78(0.69−4.59)	0.233	
		NLR_Group=Q4	1.8(0.72−4.46)	0.207	
Young	629(50.5%)	NLR_Group=Q1			
		NLR_Group=Q2	1.06(0.47−2.38)	0.895	
		NLR_Group=Q3	1.27(0.58−2.78)	0.544	
		NLR_Group=Q4	2.61(1.25−5.45)	0.011	

C

Subgroup	Event	Levels	OR.(95%CI)	P.value	P.for.interaction
**Overall**	**1246(100%)**				
**Gender**					**0.157**
Male	635(51%)	NMLR_Group=Q1			
		NMLR_Group=Q2	1(0.43−2.3)	0.995	
		NMLR_Group=Q3	0.98(0.44−2.19)	0.967	
		NMLR_Group=Q4	1.24(0.59−2.64)	0.569	
Female	611(49%)	NMLR_Group=Q1			
		NMLR_Group=Q2	0.64(0.24−1.72)	0.377	
		NMLR_Group=Q3	2.18(0.99−4.81)	0.053	
		NMLR_Group=Q4	2.06(0.91−4.68)	0.085	
**Race**					**0.75**
Mexican American	371(29.8%)	NMLR_Group=Q1			
		NMLR_Group=Q2	0.83(0.24−2.82)	0.766	
		NMLR_Group=Q3	1.92(0.62−5.94)	0.26	
		NMLR_Group=Q4	2.06(0.66−6.38)	0.212	
Other Hispanic	55(4.4%)	NMLR_Group=Q1			
		NMLR_Group=Q2	0(0−Inf)	0.996	
		NMLR_Group=Q3	1.63(0.13−20.53)	0.705	
		NMLR_Group=Q4	0.39(0.02−6.57)	0.517	
non-Hispanic White	488(39.2%)	NMLR_Group=Q1			
		NMLR_Group=Q2	0.91(0.3−2.76)	0.866	
		NMLR_Group=Q3	1.32(0.48−3.65)	0.586	
		NMLR_Group=Q4	1.4(0.52−3.75)	0.508	
non-Hispanic Black	286(23%)	NMLR_Group=Q1			
		NMLR_Group=Q2	1.51(0.45−5.06)	0.502	
		NMLR_Group=Q3	1.52(0.48−4.78)	0.474	
		NMLR_Group=Q4	0.94(0.23−3.87)	0.935	
Others	46(3.7%)	NMLR_Group=Q1			
		NMLR_Group=Q2	0(0−Inf)	0.997	
		NMLR_Group=Q3	0(0−Inf)	0.998	
		NMLR_Group=Q4	1.29(0.02−66.99)	0.898	
**Marry**					**0.491**
yes	735(59%)	NMLR_Group=Q1			
		NMLR_Group=Q2	0.68(0.29−1.61)	0.383	
		NMLR_Group=Q3	1.09(0.49−2.38)	0.838	
		NMLR_Group=Q4	1.74(0.85−3.58)	0.129	
no	511(41%)	NMLR_Group=Q1			
		NMLR_Group=Q2	1.06(0.41−2.7)	0.906	
		NMLR_Group=Q3	1.98(0.87−4.49)	0.101	
		NMLR_Group=Q4	1.45(0.6−3.48)	0.407	
**Hypertension**					**0.801**
Hypertension	828(66.5%)	NMLR_Group=Q1			
		NMLR_Group=Q2	0.71(0.32−1.57)	0.394	
		NMLR_Group=Q3	1.49(0.77−2.9)	0.239	
		NMLR_Group=Q4	1.42(0.72−2.77)	0.308	
Health	418(33.5%)	NMLR_Group=Q1			
		NMLR_Group=Q2	1.1(0.38−3.15)	0.859	
		NMLR_Group=Q3	1.44(0.5−4.16)	0.502	
		NMLR_Group=Q4	2.3(0.87−6.06)	0.092	
**Coronary heart disease(CHD)**					**0.363**
CHD	148(11.9%)	NMLR_Group=Q1			
		NMLR_Group=Q2	0.15(0.01−2.24)	0.167	
		NMLR_Group=Q3	1.07(0.24−4.69)	0.931	
		NMLR_Group=Q4	1.13(0.26−4.92)	0.867	
Health	1098(88.1%)	NMLR_Group=Q1			
		NMLR_Group=Q2	0.99(0.51−1.91)	0.973	
		NMLR_Group=Q3	1.5(0.81−2.76)	0.197	
		NMLR_Group=Q4	1.62(0.89−2.95)	0.118	
**Heart failure(HF)**					**0.107**
Heart failure	158(12.7%)	NMLR_Group=Q1			
		NMLR_Group=Q2	0.28(0.03−3.07)	0.298	
		NMLR_Group=Q3	2.64(0.62−11.36)	0.191	
		NMLR_Group=Q4	2.78(0.67−11.48)	0.158	
Health	1088(87.3%)	NMLR_Group=Q1			
		NMLR_Group=Q2	0.99(0.51−1.91)	0.972	
		NMLR_Group=Q3	1.3(0.7−2.41)	0.403	
		NMLR_Group=Q4	1.42(0.78−2.61)	0.254	
**Age**					**0.258**
Old	617(49.5%)	NMLR_Group=Q1			
		NMLR_Group=Q2	1.38(0.52−3.69)	0.518	
		NMLR_Group=Q3	1.69(0.66−4.36)	0.275	
		NMLR_Group=Q4	1.68(0.67−4.16)	0.266	
Young	629(50.5%)	NMLR_Group=Q1			
		NMLR_Group=Q2	0.54(0.22−1.33)	0.183	
		NMLR_Group=Q3	1.41(0.69−2.9)	0.345	
		NMLR_Group=Q4	2.06(1.01−4.22)	0.048	

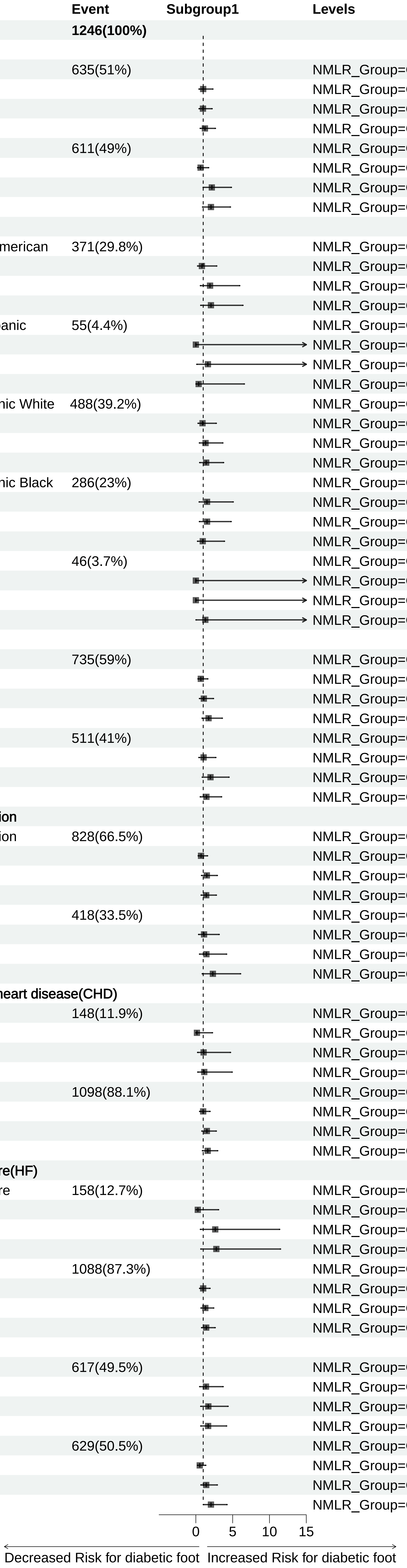

D

Subgroup	Event	Subgroup1	Levels	OR.(95%CI)	P.value	P.for.interaction
**Overall**	**1246(100%)**					
**Gender**						**0.726**
Male	635(51%)		SIRI_Group=Q1			
			SIRI_Group=Q2	0.66(0.27−1.65)	0.374	
			SIRI_Group=Q3	1.37(0.64−2.93)	0.417	
			SIRI_Group=Q4	1.27(0.59−2.72)	0.538	
Female	611(49%)		SIRI_Group=Q1			
			SIRI_Group=Q2	0.84(0.36−1.96)	0.693	
			SIRI_Group=Q3	1.04(0.45−2.42)	0.926	
			SIRI_Group=Q4	1.64(0.75−3.6)	0.215	
**Race**						**0.285**
Mexican American	371(29.8%)		SIRI_Group=Q1			
			SIRI_Group=Q2	0.71(0.22−2.25)	0.557	
			SIRI_Group=Q3	1.79(0.66−4.81)	0.251	
			SIRI_Group=Q4	1.62(0.56−4.69)	0.373	
Other Hispanic	55(4.4%)		SIRI_Group=Q1			
			SIRI_Group=Q2	1.37(0.16−11.85)	0.774	
			SIRI_Group=Q3	0(0−Inf)	0.996	
			SIRI_Group=Q4	0.49(0.02−15.23)	0.681	
non-Hispanic White	488(39.2%)		SIRI_Group=Q1			
			SIRI_Group=Q2	0.94(0.26−3.38)	0.919	
			SIRI_Group=Q3	1.64(0.51−5.3)	0.407	
			SIRI_Group=Q4	1.82(0.59−5.63)	0.301	
non-Hispanic Black	286(23%)		SIRI_Group=Q1			
			SIRI_Group=Q2	0.58(0.17−1.98)	0.384	
			SIRI_Group=Q3	0.87(0.28−2.73)	0.815	
			SIRI_Group=Q4	0.5(0.1−2.51)	0.401	
Others	46(3.7%)		SIRI_Group=Q1			
			SIRI_Group=Q2	0(0−Inf)	0.998	
			SIRI_Group=Q3	0(0−Inf)	0.998	
			SIRI_Group=Q4	4.5(0.13−151.65)	0.402	
**Marry**						**0.378**
yes	735(59%)		SIRI_Group=Q1			
			SIRI_Group=Q2	0.47(0.18−1.2)	0.115	
			SIRI_Group=Q3	1.22(0.59−2.54)	0.591	
			SIRI_Group=Q4	1.58(0.77−3.22)	0.211	
no	511(41%)		SIRI_Group=Q1			
			SIRI_Group=Q2	1.08(0.46−2.56)	0.856	
			SIRI_Group=Q3	1.28(0.55−3)	0.572	
			SIRI_Group=Q4	1.27(0.55−2.94)	0.572	
**Hypertension**						**0.611**
Hypertension	828(66.5%)		SIRI_Group=Q1			
			SIRI_Group=Q2	0.66(0.31−1.4)	0.282	
			SIRI_Group=Q3	0.95(0.47−1.9)	0.881	
			SIRI_Group=Q4	1.31(0.68−2.5)	0.423	
Health	418(33.5%)		SIRI_Group=Q1			
			SIRI_Group=Q2	0.91(0.3−2.74)	0.873	
			SIRI_Group=Q3	2.17(0.85−5.53)	0.106	
			SIRI_Group=Q4	1.8(0.67−4.85)	0.247	
**Coronary heart disease(CHD)**						**0.065**
CHD	148(11.9%)		SIRI_Group=Q1			
			SIRI_Group=Q2	4811204.29(0−Inf)	0.992	
			SIRI_Group=Q3	20029549.31(0−Inf)	0.991	
			SIRI_Group=Q4	29015719.22(0−Inf)	0.991	
Health	1098(88.1%)		SIRI_Group=Q1			
			SIRI_Group=Q2	0.7(0.37−1.32)	0.269	
			SIRI_Group=Q3	1.09(0.61−1.97)	0.764	
			SIRI_Group=Q4	1.13(0.63−2.02)	0.687	
**Heart failure(HF)**						**0.543**
Heart_failure	158(12.7%)		SIRI_Group=Q1			
			SIRI_Group=Q2	0.81(0.1−6.46)	0.844	
			SIRI_Group=Q3	1.83(0.33−9.97)	0.487	
			SIRI_Group=Q4	3.75(0.75−18.7)	0.107	
Health	1088(87.3%)		SIRI_Group=Q1			
			SIRI_Group=Q2	0.76(0.4−1.45)	0.406	
			SIRI_Group=Q3	1.24(0.68−2.24)	0.487	
			SIRI_Group=Q4	1.18(0.65−2.14)	0.586	
**Age**						**0.605**
Old	617(49.5%)		SIRI_Group=Q1			
			SIRI_Group=Q2	0.88(0.34−2.23)	0.782	
			SIRI_Group=Q3	0.98(0.4−2.45)	0.97	
			SIRI_Group=Q4	1.49(0.65−3.41)	0.345	
Young	629(50.5%)		SIRI_Group=Q1			
			SIRI_Group=Q2	0.63(0.27−1.48)	0.291	
			SIRI_Group=Q3	1.56(0.77−3.16)	0.216	
			SIRI_Group=Q4	1.51(0.72−3.2)	0.278	

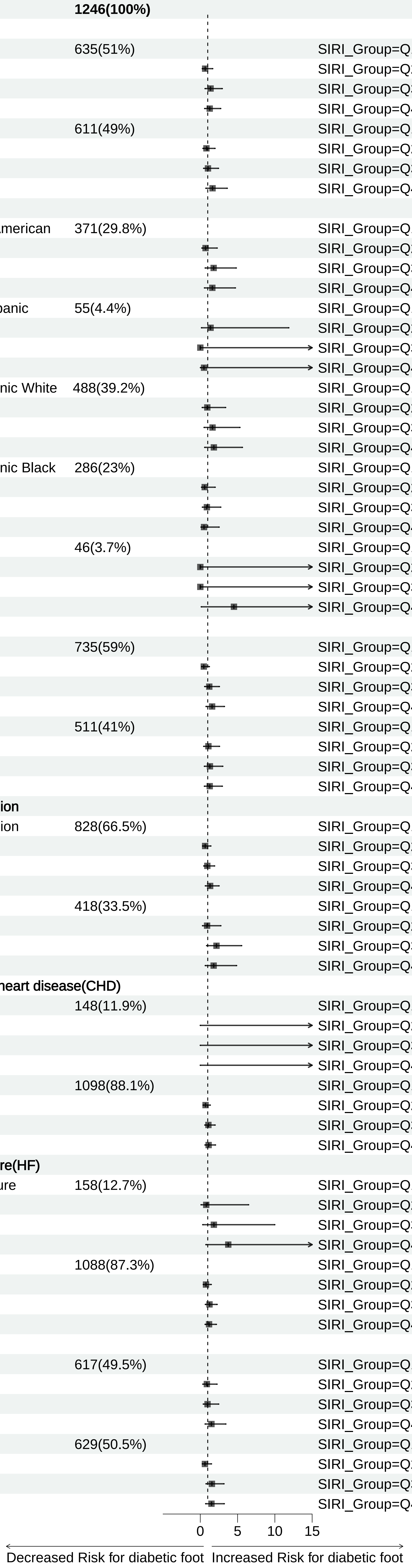

Supplement: S1 Fig — (PDF) [file pone.0326082.s001.pdf]
