# Supplementary material for: Are metabolic abnormalities the missing link between complete blood count-derived inflammatory markers and diabetic foot? Evidence from a large population study
Source: PLoS One. 2025 Jun 12;20(6):e0326082. doi: 10.1371/journal.pone.0326082 (PMC12161541; doi:10.1371/journal.pone.0326082)
Supplement: S1 Table — (DOCX) [file pone.0326082.s002.docx]

| S1 Table. Analysis of the mediation by Metabolic related indicators of the associations of CBC-Derived inflammatory markers | | | | |
| --- | --- | --- | --- | --- |
|  |  | Mediation effect (95% CI), P value |  |  |
|  | Total effect | Direct effect | Indirect effect | Mediation |
| MLR |  |  |  |  |
| Alb | 0.19(0.08,0.3)<0.001 | 0.16(0.07,0.28)<0.001 | 0.02(0.01,0.04)<0.001 | 11.33% |
| Cr | 0.19(0.09,0.3)<0.001 | 0.16(0.06,0.28)<0.001 | 0.02(0.01,0.04)<0.001 | 12.46% |
| RDW | 0.18(0.07,0.27)<0.001 | 0.16(0.05,0.26)<0.001 | 0.02(-0.01,0.04)0.2 | 11.14% |
| NLR |  |  |  |  |
| Alb | 0.02(0,0.03)0.04 | 0.01(0,0.02)0.04 | 0(0,0)<0.001 | 13.02% |
| Cr | 0.02(0,0.03)<0.001 | 0.01(0,0.03)0.04 | 0(0,0)<0.001 | 8.73% |
| RDW | 0.02(0.01,0.03)<0.001 | 0.02(0,0.03)0.04 | 0(0,0)0.24 | 8.26% |
| NMLR |  |  |  |  |
| Alb | 0.02(0,0.02)0.04 | 0.01(0,0.02)0.04 | 0(0,0)0.04 | 11.83% |
| Cr | 0.02(0.01,0.03)0 | 0.02(0,0.02)<0.001 | 0(0,0)<0.001 | 8.81% |
| RDW | 0.02(0.01,0.03)0 | 0.02(0.01,0.03)<0.001 | 0(0,0)0.24 | 10.01% |
| SIRI |  |  |  |  |
| Alb | 0.03(0.01,0.04)0 | 0.03(0.01,0.04)<0.001 | 0(0,0)<0.001 | 8.72% |
| Cr | 0.03(0.01,0.04)0 | 0.03(0.01,0.04)<0.001 | 0(0,0)0.12 | 5.47% |
| RDW | 0.03(0.02,0.04)0 | 0.03(0.02,0.04)<0.001 | 0(0,0.01)0.24 | 8.36% |
| Abbreviations: RDW Red blood cell distribution width, ALB albumin, Cr creatinine | | | | |
