# Supplementary material for: Are metabolic abnormalities the missing link between complete blood count-derived inflammatory markers and diabetic foot? Evidence from a large population study
Source: PLoS One. 2025 Jun 12;20(6):e0326082. doi: 10.1371/journal.pone.0326082 (PMC12161541; doi:10.1371/journal.pone.0326082)
Supplement: Attachment — Submitted filename: Comments to the Authors.docx [file pone.0326082.s003.docx]

The study addresses an important medical issue—diabetic foot and its relationship with inflammatory markers—which has significant clinical implications.

NHANES data enhances the study’s reliability and generalizability.

The inclusion of multiple inflammatory markers, statistical models, subgroup analysis, and mediation analysis strengthens the depth of investigation.

The study design, inclusion/exclusion criteria, and statistical methods are well-explained, making the research reproducible.

The findings may contribute to early detection and intervention strategies for diabetic foot.

**Abstract:**

o	While generally well-structured, it should be more concise. The description of statistical methods could be streamlined to avoid overwhelming the reader.

o	Conclusion should reinforce the clinical significance of the findings rather than simply restating them.

**Introduction**

o	The background is informative but could be more engaging. Consider starting with a strong statement on the burden of diabetic foot rather than statistics.

o	The rationale for the study could be framed more clearly. While the study highlights gaps in previous research, a more explicit statement on why this study is novel would improve the introduction.

**Materials & Methods**

o	The study design is well-detailed but somewhat dense, sections could be made more concise to enhance readability (e.g., reducing redundancy in technical terms).

**Discussion**

•	The discussion could benefit from a brief interpretation of why these metabolic markers mediate the relationship between CBC-derived inflammatory markers and diabetic foot. Do these biomarkers reflect a specific pathophysiological mechanism? A short statement on this would improve the theoretical depth of the analysis.

•	Consider adding a brief concluding remark summarizing the implications of the results. This would help tie together the statistical findings with their broader clinical or epidemiological significance.

•	The section on the Restricted Cubic Spline (RCS) method could briefly explain why it was chosen over other non-linear modeling approaches. What specific advantages does it offer in this study?

•	Going deeper into the correlation between the most significant analyzed parameters and their relationship with diabetic foot could enrich the discussion, also specifying the mechanisms of possible biases (correlation with other concomitant diseases) to provide a starting point for future similar analyses

**Conclusion**

Emphasize clinical implications of findings can make a more impactful conclusion.
